# Bilateral Vertebral Artery Hypoplasia and Fetal-Type Variants of the Posterior Cerebral Artery in Acute Ischemic Stroke

**DOI:** 10.3389/fneur.2021.582149

**Published:** 2021-04-08

**Authors:** Chung-Fu Hsu, Kuan-Wen Chen, Chun-Hung Su, Chao-Yu Shen, Hsin-Yi Chi

**Affiliations:** ^1^Department of Neurology, Chung Shan Medical University Hospital, Taichung, Taiwan; ^2^Department of Neurology, Chung Shan Medical University, Taichung, Taiwan; ^3^Institute of Medicine, School of Medicine, Chung Shan Medical University, Taichung, Taiwan; ^4^Division of Cardiology, Department of Internal Medicine, Chung Shan Medical University Hospital, Taichung, Taiwan; ^5^Department of Medical Imaging, Chung Shan Medical University Hospital, Taichung, Taiwan; ^6^College of Medicine, Pharmacological Institute, National Taiwan University, Taipei, Taiwan

**Keywords:** posterior circulation infarction, vertebral artery hypoplasia, posterior cerebral artery, vertebrobasilar insufficiency, ultrasonagraphy

## Abstract

**Aim:** Unilateral vertebral artery hypoplasia is considered a risk factor for posterior circulation infarction. Despite the increasing attention on unilateral vertebral artery hypoplasia, few studies have discussed bilateral vertebral artery hypoplasia, its influence on stroke, or its collateral supply from the circle of Willis. We aimed to identify its characteristics, stroke pattern, and unique ultrasonographic and brain imaging findings.

**Materials and Methods:** Of the 1,301 consecutive in-patients diagnosed with acute ischemic stroke from January 2013 to December 2015, medical and laboratory data and stroke or transient ischemic attack history were recorded. We enrolled patients who underwent both brain magnetic resonance imaging and sonography examinations. Vertebral artery and posterior cerebral artery analyses were conducted in accordance with clinical criteria.

**Results:** Adequate imaging data were available for 467 patients. Of these, eight patients met the criteria for bilateral vertebral artery hypoplasia. The mean age was 62.9 ± 12.1 years. There were six male (75.0%) and two female patients (25.0%). A high prevalence of hypertension (7/8, 87.5%) was noted.

Sonograms displayed a very low net flow volume in the vertebral arteries, with the average net flow volume being 28.9 ± 9.7 mL/min. A high frequency (6/8; 75.0%) of the fetal variant posterior cerebral artery from the carotids was found. The infarction patterns in these patients were all bilateral, scattered, and in multiple vascular territories.

**Conclusion:** Patients with bilateral vertebral hypoplasia displayed a unique collateral supply, special stroke pattern, and younger stroke onset. Early recognition and stroke prevention should be considered critical in clinical practice.

## Introduction

Posterior circulation is comprised of two vertebral arteries that join to form a single basilar artery at the level of the pons. The basilar artery divides into two posterior cerebral arteries at the level of the midbrain (1–3). Conventionally, the major hemodynamics of this area is supplied by the net flow of the vertebral arteries and partially by the collateral flow from the spinal arteries or the fetal-type posterior cerebral artery (1, 4).

Case series studies suggest that vertebral artery hypoplasia (VAH) may contribute to posterior ischemic events, especially in patients with other cerebrovascular risk factors (5, 6). The concept of regional hypoperfusion is associated with unilateral VAH and posterior circulation stroke (7). The risk of posterior ischemia is related to an increasing degree of VAH (5, 6, 8), regardless of the net flow (7). Although an increasing number of studies highlight the importance of unilateral VAH on ischemic stroke (5, 6, 8, 9), literature discussing the influence of bilateral VAH on ischemic stroke is limited (10, 11).

With regard to bilateral VAH, low flow volume in a single vertebral artery as well as inadequate net flow volume in the basilar artery ensues. Because of the chronic nature of congenital hypoplasia, the clinical symptoms and stroke patterns of this vascular disorder would differ from unilateral VAH. Chronic inadequate posterior circulation leads to the development of intracranial and extracranial collateral flow (10, 12). Recently, a case series paper correlated the fetal-type posterior circle of Willis with vertebrobasilar hypoplasia (12, 13).

In order to identify obscure clinical features, we reviewed the characteristics of patients with bilateral VAH by analyzing their clinical presentations, stroke patterns, risk factors, and the hemodynamics of collateral flow using ultrasonography and brain magnetic resonance imaging (MRI).

## Materials and Methods

### Patients

This is a retrospective, observational cross-sectional study. We reviewed 1,301 consecutive in-patients diagnosed with acute ischemic stroke at the Chung Shan Medical University Hospital from January 2013 to December 2015.

Upon admission, the series examination included an MRI, sonography exam, and a stroke risk factor survey. Patients who did not receive a brain MRI or sonography exam were excluded.

Patient medical and laboratory data were recorded; this included the age, sex, presence of systemic diseases, renal function, lipid profile, drug use, electrocardiogram, and history of previous stroke or transient ischemic attacks and related clinical manifestations.

This study was approved by the Institutional Review Board of Chung Shan Medical University Hospital, Taichung, Republic of China.

### MRI

The infarction lesions were identified and classified by vascular territory according to MRI and three-dimensional time of flight (3D TOF) magnetic resonance angiography (MRA) examinations. A 3-T MRI system (Siemens, Germany) with the following settings was used T2-weightedimages, TR/TE 6000/100 ms, diffusion-weighted images TR/TE 5100/60 ms, and 3D TOF TR/TE20/4 ms. The locations of ischemic stroke were categorized as proximal (medulla and posterior inferior cerebellum), middle (pons and anterior inferior cerebellum), and distal (rostral brainstem, superior cerebellum, and occipital and temporal lobes) intracranial posterior circulation territories (14, 15).

A fetal type posterior cerebral artery (PCA) was classified as complete or partial according to MRA (12, 13). A complete fetal-type PCA is considered if the P1 segment is not visualized, and a partial fetal-type PCA is considered if the P1 segment is smaller than the posterior communicating artery on brain MRI.

The basilar artery hypoplasia (BAH) was defined as a basilar artery (BA) diameter <2 mm. The BA diameter was calculated on TOF source images at the mid-pons level. Vascular dissection was diagnosed if intramural hematoma, intimal flap, the pearl-and-string, or the double lumen signs were visualized on MRI.

Brain images were reviewed by neuroradiologists and neurologists, with the former interpreting the fetal-type PCA.

### Sonography

Color-coded carotid duplex and transcranial color-coded duplex examinations were reviewed for all enrolled patients. Intracranial and extracranial vessels conducted by experienced technicians using an IE-33 system (Philips Medical System, USA), equipped with a 2.0-MHz transducer. Routine measurements included thorough examinations of the bilateral neck carotid and transforaminal windows. The angle between the ultrasound beam and the direction of blood flow was adjusted manually. Blood flow examinations were targeted at the V2 and V4 segments of the vertebral artery as well as the region proximal to the distal basilar artery. The diameter and flow volume of each extracranial vertebral artery, as well as the mean velocity, and pulsatility index of the intracranial vertebrobasilar arteries were recorded and analyzed. VAH was defined according to sonographic criteria (4, 16, 17), including a ≤2.2 mm diameter over the V2 segment or a decreased vertebral flow volume of ≤30 mm/s. Bilateral VAH was defined as both vertebral arteries meeting the sonographic criteria of VAH and there was no evidence of dissection findings on MRI.

### Statistics

All statistical analyses were performed using SPSS software (version 22.0; SPSS Inc., Chicago, IL, USA). Continuous data is expressed as mean±SD.

## Results

Of the 1,301 patients diagnosed with acute ischemic stroke, 467 (149 pateints with posterior circulation infarction) underwent both MRI and sonographic examinations and were enrolled in the present study. In patients with posterior circulation, 80 of them met the criteria of unilateral VAH. Eight of the enrolled patients met the diagnostic criteria for bilateral VAH. The mean BA diameter was 2.68 ± 0.20 mm (range from 2.29 to 3.08 mm). In our study, the frequency of bilateral VAH was 1.7% (8/467), which is similar to that found in previous studies (11, 18). The characteristics of patients with bilateral VAH are listed in Table 1. There were six male (75.0%) and two female patients (25.0%). The average age was 62.9 years (range, 46–87). None of these patients had atrial fibrillation or heart disease. A high prevalence of hypertension (7/8, 87.5%) was noted. The prevalence of other stroke risk factors, including diabetes mellitus, dyslipidemia, smoking, and alcohol consumption ranged from 12.5 to 25.0%. We compared net flow volume, onset age, and stroke location analysis in bilateral VAH, unilateral VAH, and non-VAH groups, listed in Table 2. The mean net flow volume was 28.9 ± 9.7 mL/min, which is below the criteria of vertebrobasilar insufficiency (<100 mL/min) (7), and the defined value of unilateral VAH (30 mL/min) (16). In the bilateral VAH group, we found their onset age was younger and with more multiple vascular territory lesions.

**Table 1:** Demographic and clinical characteristics of eight patients with bilateral vertebral artery hypoplasia.

	**Bilateral hypoplasia (*n* = 8)**	**Range**
Age on stroke, years, mean ±SD	62.9 ± 12.1	46–87
Gender(male), n (%)	6 (75%)	
Hypertension, n (%)	7 (87.5%)	
Diabetes, n (%)	2 (25%)	
Hyperlipidemia, n (%)	1 (12.5%)	
Smoking, n (%)	2 (25%)	
Alcohol, n (%)	2 (25%)	
Renal function, eGFR(mg/dL)	77.3 ± 25.8	29–119
Atrial fibrillation or heart disease, n (%)	0 (0%)	

*SD, standard deviation; Egfr, estimated glomerular filtration rate*.

**Table 2:** Total VA flow volume, onset age and stroke location analysis in bilateral, unilateral, or non-vertebral artery hypoplasia groups.

	**Non-VAH (61 cases)**	**Unilateral-VAH (80 cases)**	**Bilateral-VAH (8 cases)**
Total VA flow volume	132.9 ± 31.8 mL/min	71.4 ± 21.1 mL/min	28.9 ± 9.7 mL/min
Stroke onset age	66.6 ± 11.5	72.4 ± 10.2	62.9 ± 12.9
Multiple vascular	9/61 (14.7%)	17/80 (21.2%)	5/8 (62.5%)
territory infarctions			

*VAH, vertebral artery hypoplasia; VA, vertebral artery*.

The distribution of stroke and individual PCA types is listed in Table 3. Most of the infarctions were bilateral and multiple (5/8, 62.5%). A fetal-type PCA was recognized in six patients (6/8, 75.0 %), two with a complete bilateral, two with a partial bilateral, and two with a complete unilateral fetal-type PCA.

**Table 3:** Infarction region and clinical manifestation of stroke in patient population.

**Age**	**Gender**	**Infarction territory**	**Brain territory locations**	**PCA type**	**Initial manifestation at stroke**
41–50	Male (Patient 1)	Pons, midbrain and bilateral cerebellum hemisphere	^*^P+M+D	Bilateral fetal type	Dizziness and unsteady gait
	Male (Patient 2)	Left PICA territory (lateral medulla)	P	Bilateral partial fetal type	Right limb paresthesia
51–60	Male (Patient 3)	Right medulla, pons, and cerebellum	P+M	Right fetal type	Dizziness, diplopia and left facial paresthesia
	Female (Patient 4)	Left pons and bilateral cerebellum hemisphere	P+M+D	Bilateral partial fetal type	Dizziness and unsteady gait
61–70	Male (Patient 5)	Left pons and left anterior medulla and right vermis	P+M+D	Non-fetal type	Right hemiparesis and unsteady gait, lethargy
	Male (Patient 6)	Left thalamus and left occipital lobe	D	Right fetal type	Blurred vision and right hemiparesis and right paresthesia
71–80	Male (Patient 7)	Bilateral pons (right>left), midbrain, and bilateral cerebellar hemisphere	P+M+D	Non-fetal type	Severe dysarthria and left hemiplegia
81–90	Female (Patient 8)	Bilateral pons and cerebellar hemisphere	M+D	Bilateral fetal type	Dysarthria, dysphagia, and unsteady gait

* *P, proximal; M, middle; D, distal posterior circulation territory*.

## Discussion

The prevalence of VAH ranges from 4 to 7% in the normal population (6, 7, 17). In posterior circulation stroke, the prevalence is increased to more than 40% according to different clinical studies (5, 18). Bilateral VAH is recognized in 1.6 to 3.4 % of patients with ischemic stroke (9, 18).

In our previous study, compared to anterior circulation infarction, there was a significantly higher frequency of VAH in posterior circulation infarction (22.38 vs. 44.75%, *p* < 0.0001) (7). Literature has demonstrated that VAH plays an important role in posterior circulation stroke (5–7, 9).

In this study, we found that patients with bilateral VAH developed stroke at a younger age (six patients <66 years) than the mean age of ischemic stroke, which ranges from 66 to 70 years (19), and other groups with posterior circulation infarctions (Table 2). The prevalence of hypertension in this group was significantly higher than the general stroke population (20). Cerebral autoregulation for chronic vertebrobasilar insufficiency may explain the blood pressure response (21).

**Figure 1:**
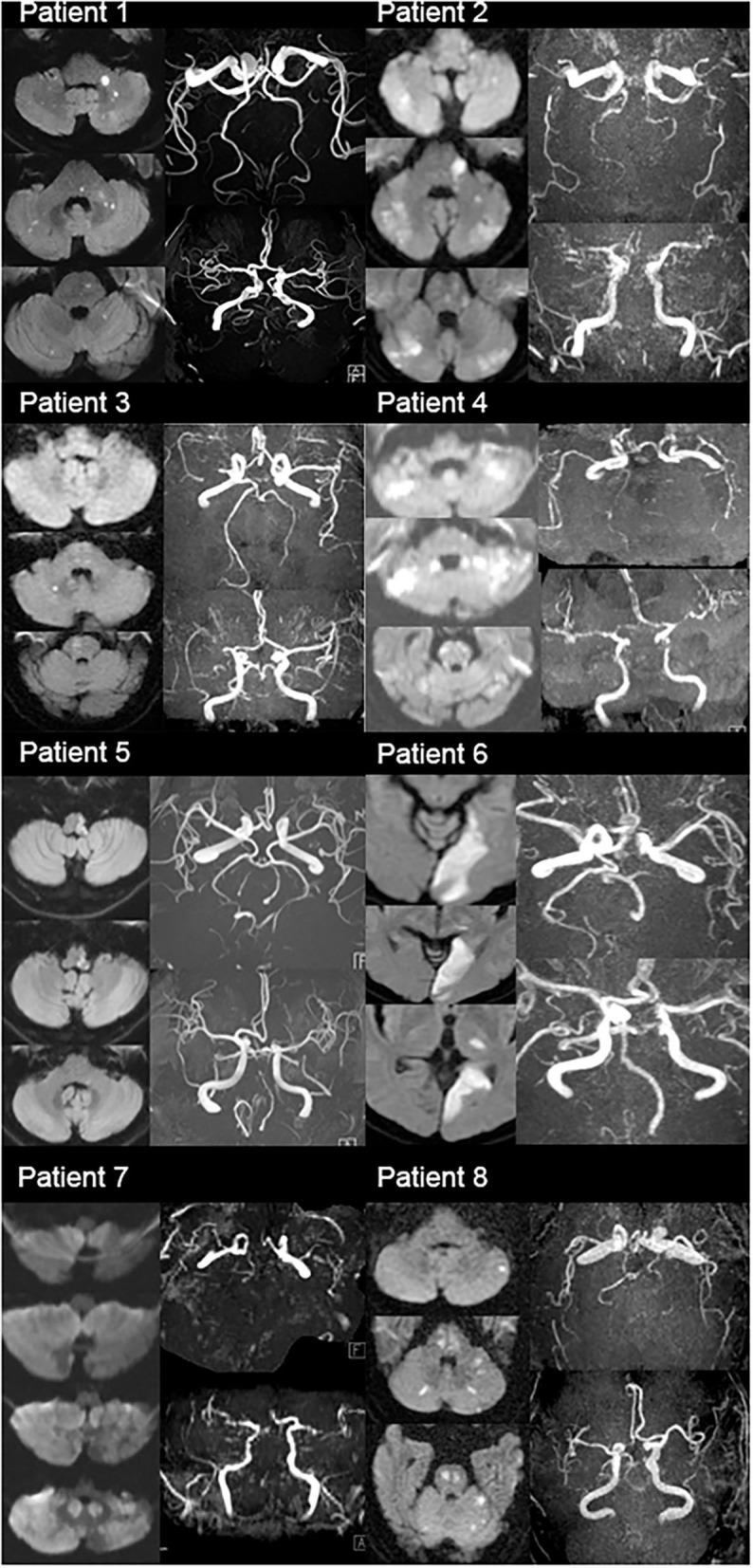
MRI diffusion-weighted images (DMI) and MRA images of total eight patients (MRI DWI showed multiple infarctions in these patients; MRA showed fetal type PCA, vertebral hypoplasia, small caliber of basilar artery, or invisible vertebrobasilar arteries).

Table 3 displays the initial manifestation of the patient population, which included dizziness, severe dysarthria, hemiplegia, and ataxia. In unilateral VAH-related stroke, the location of the infarct is usually limited to the territory of the ipsilateral artery, particularly in lateral medullar infarction and posterior inferior cerebellar artery infarction (2, 22, 23). In bilateral VAH, the infarction territory was mostly bilateral, involving multiple vascular territories, despite receiving collateral blood supply from the fetal-type PCA (Table 3 and Figure 1). The clinical presentation of these patients illustrates the high variability and burden of posterior circulation infarction. Most cases of multiple infarctions resulted in severe handicap or coma; therefore, it is important to detect at-risk patients.

Scattered brain infarction is usually related to cardioembolic stroke or artery-to- artery embolism (24); however, no patients in our population had atrial fibrillation, other heart disease, or significant atherosclerosis across the major arteries, suggesting the effect of hypoperfusion in bilateral VAH-related stroke. Several studies reported unilateral VAH to be associated with relative hypoperfusion in the dependent vascular territory (5, 7). According to the extracranial ultrasonography of our patient group, the mean (±SD) of the total vertebral flow volume was low(28.9 ± 9.7 mL/min, Table 2), compared with the non-VAH or unilateral VAH group (Table 2), which suggest severe hypoperfusion of the vertebrobasilar system, and as a consequence, development of an earlier and more severe posterior circulation infarction (Table 2).

In this study, bilateral VAH evolving into a smaller basilar artery (mean BA diameter 2.68 ± 0.20 mm, range from 2.29 to 3.08 mm) was recognized. Artery to artery embolism from vertebrobasilar hypoplasia would also contribute to scattered infarctions. Literature has demonstrated (25) BAH was associated with pontine infarction and VAH was associated with the medulla and inferior cerebellum. Emboli were known to preferentially reach the distal posterior circulation arteries (14). However, it is difficult to recognize the true origin of embolism since the artery to artery embolism and large artery hemodynamic should be one of concern.

For the treatment of bilateral VAH, early preventive drugs for ischemic insults, including antiplatelet or anticoagulant drugs, could be considered in symptomatic patients. Reconstruction of the blood supply, such as bypass surgery, would be another option (26).

Conventionally, a fetal-type PCA was thought to be a normal variant and common in the general population; however, in some reports this vascular type was associated with a higher risk for ischemic stroke, both in the anterior and posterior circulation (3, 4, 27, 28). Until now, its significance has been under debate.

In the literature, the incidence of a unilateral and bilateral fetal-type PCA ranged from 4 to 26% and 2 to 4%, respectively (13, 27, 28). Studies state commonly reported symptoms in patients with a fetal-type PCA to be dizziness, headache, and focal neurological deficits (3, 28). In our population, 75% of patients with bilateral VAH also displayed a fetal-type PCA, illustrating a sizable co-existence (75%) of bilateral VAH and a unilateral or bilateral fetal-type PCA. This finding corresponds to findings from previous studies that suggest the simultaneous occurrence of a hypoplastic vertebrobasilar system and fetal-type circle of Willis, and the increased development of ischemic events in the posterior circulation (3, 12).

From an embryological perspective, due to the delayed development of the P1 segment, the PCAs are supplied by the internal carotid arteries via the posterior communicating arteries temporally.

Typically, an adult PCA is complete at 6–7 weeks of embryological development (29). Inadequate flow of the basilar PCA system may interrupt the normal development of the PCA. Nevertheless, it is not clear how a fetal-type circle of Willis responds to unilateral VAH (30) or significantly inadequate basilar flow (in this study), or how it evolves to the adult configuration, which leads to a higher risk for both anterior and post-ischemic strokes (12, 27). However, the significance and pathophysiology of a fetal-type PCA in stroke remains unclear. Further comprehensive research is necessary.

In this study, most patients with bilateral VAH displayed a fetal variant of the PCA, supplied from the anterior circulation via the posterior communicating artery segment. However, with such hemodynamic compensation, supplementation via the fetal-type PCA still failed to support the vertebrobasilar system, resulting in a multifocal scattered infarction in the posterior circulation.

There were several limitations in this study. First, the case number of bilateral VAH is small, and we enrolled our samples from in-patients and not in healthy subjects. Overestimated frequencies and symptoms of bilateral VAH would be suspected. Because we applied the duplex ultrasonographic criteria of VAH, stenosis over the VA orifice or decreased VA flow volume due to atherosclerosis stenosis is a possible trap as applying the sonographic criteria of VAH diagnosis. Compared evaluation of the contrast-enhanced MRA images at the same time would result in a more reliable diagnosis.

## Conclusion

We evaluated the clinical and vascular characteristics of patients with stroke and bilateral VAH. We found a younger age at stroke onset, obvious hypertension, bilateral and multiple vertebrobasilar infarcts, and a high prevalence of the fetal PCA in our enrolled patients.

Clinically, bilateral VAH may pose a significant risk to the posterior circulation; therefore, early detection and prevention are crucial in this patient group.

## Data Availability Statement

The raw data supporting the conclusions of this article will be made available by the authors, without undue reservation.

## Ethics Statement

The studies involving human participants were reviewed and approved by Institutional Review Board of Chung Shan Medial University Hospital, Taichung, Republic of China. The patients/participants provided their written informed consent to participate in this study.

## Author Contributions

H-YC designed the study. C-FH, K-WC, C-HS, and C-YS collected and organized data. C-FH and H-YC analyzed and interpreted the data. All authors read and approved the final manuscript.

## Conflict of Interest

The authors declare that the research was conducted in the absence of any commercial or financial relationships that could be construed as a potential conflict of interest.
